# How Healthcare Interactions Contribute to Burden for Care Partners of Older Adults

**DOI:** 10.1093/geroni/igaa057.492

**Published:** 2020-12-16

**Authors:** Anne Mueller, Beth Fields

**Affiliations:** University of Wisconsin–Madison, Madison, Wisconsin, United States

## Abstract

As the aging population in the U.S. continues to grow, care partners (i.e. family and friends) are assuming increasingly intense and complex caregiving responsibilities. Care partner burden is associated with poorer health outcomes for older adults and more frequent rehospitalizations. This secondary data analysis aims to examine the relationship between different types of health care interactions and care partner burden. A total of 2,588 care partners of Medicare beneficiaries age 65 and older were included. Secondary analyses were conducted using cross-sectional data from the 2017 National Study of Caregiving. Logistic regression analyses were used to determine the relationship between health care interactions and care partner burden while controlling for demographic characteristics. The average care partner was 62 years old (range 18-98), female (68.1%), and white (62.8%). More than half of the care partners (51.3%) reported financial, emotional, and/or physical difficulty as a result of helping the older adult. Logistic regression analyses show that care partners who made medical appointments (AOR=2.04), accessed online medical information (AOR=1.55), and coordinated care between medical providers (AOR=2.15) were significantly more likely to report burden. Care partners are important allies in supporting the health of older adults but may experience excess burden due to health care interactions. Practitioners and researchers may need to evaluate ways to improve the ease, efficiency, and accessibility of different types of health care interactions for care partners of older adults. A better understanding of factors that contribute to care partner burden may inform tailored interventions and future health and aging policies.

